# Comparison of Targeted Axillary Dissection with Sentinel Node Biopsy Alone on Nodal Recurrence for Patients who have Node-Positive Breast Cancer Treated with Neoadjuvant Chemotherapy

**DOI:** 10.1245/s10434-025-17197-w

**Published:** 2025-03-25

**Authors:** Marissa K. Boyle, Farin Amersi, Alice Chung, Joshua Tseng, Armando E. Giuliano

**Affiliations:** Division of Surgical Oncology, Department of Surgery, Cedars-Sinai Medical Center, Los Angeles, CA USA

**Keywords:** Targeted axillary dissection, Sentinel lymph node biopsy, Breast cancer, Neoadjuvant chemotherapy

## Abstract

**Background:**

For patients with node-positive breast cancer whose axilla is clinically downstaged after neoadjuvant chemotherapy (NAC), targeted axillary dissection (TAD) has been adopted at several institutions. This study compared axillary nodal recurrence between TAD and sentinel lymph node biopsy (SLNB) alone.

**Methods:**

Consecutive patients with stage II or III biopsy-proven node-positive breast cancer treated with NAC from August 2018 to June 2022 were identified. Patients who became clinically node-negative after NAC and had tumor-free SLNB were evaluated. The patients were divided into two groups: the TAD and SLNB-alone groups.

**Results:**

Of the 377 patients treated with NAC, 143 (38 %) had stage II or III node-positive breast cancer, 105 (73 %) were converted to ycN0, and 44 (42 %) had tumor-free SLNB and avoided an axillary lymph node dissection (ALND). Of the 44 patients, 25 (57 %) had TAD, and 19 (43 %) had SLNB alone. The TAD and SLNB-alone groups were clinically similar. The median tumor size was 2.7 cm (range, 1.9–3.4 cm). The SLNB-alone approach was less likely to retrieve the biopsy-proven clipped node (clipped node retained: overall [*n* = 5/37], TAD [*n* = 1], SLNB alone [*n* = 4]; *p* = 0.03). Adjuvant radiotherapy (RT) was administered to 40 patients (91 %) and regional nodal RT to 32 patients (73 %). During a median follow-up period of 28 months, no axillary nodal recurrences were found in either group.

**Conclusions:**

For the patients with stage II or III node-positive breast cancer who became cN0 after NAC, with tumor-free sentinel nodes, axillary nodal recurrence rates were low after both TAD and SLNB alone despite rates of higher non-retrieval of the clipped node in the SLNB-alone group. These findings suggest that either method affords excellent staging and regional control.

For patients with node-positive breast cancer whose axilla is clinically downstaged after neoadjuvant chemotherapy (NAC), prospective trials demonstrate an acceptable staging false-negative rate with sentinel lymph node biopsy (SLNB).^1–4^ Frequently, NAC allows for eradication of lymph node disease, reaching the goal of achieving an axillary pathologic complete response (pCR).

Different methods are used for surgical evaluation of the axilla for residual tumor burden after NAC. Performance of SLNB using a dual tracer and achieving three or more sentinel nodes reduces the false-negative rate to an acceptable threshold of less than 10 %. Additional studies have demonstrated low axillary recurrence with use of this method.^5^

A limitation of SLNB alone is that the biopsy-proven metastasis may not be a sentinel lymph node at the time of surgery after NAC and may be retained. The clinical significance of retained biopsy-proven metastasis is unknown. Because of this limitation, several institutions have adopted targeted axillary dissection (TAD), which is the surgical technique of SLNB with selective localization and removal of biopsy-proven metastasis.^6^ Although emerging data also have demonstrated low axillary recurrence after SLNB alone,^7^ data comparing the methods of TAD and SLNB alone are limited.^8^ This study compared axillary nodal recurrence between patients who had TAD and those who underwent SLNB alone. (Fig. 1)

**Fig. 1:**
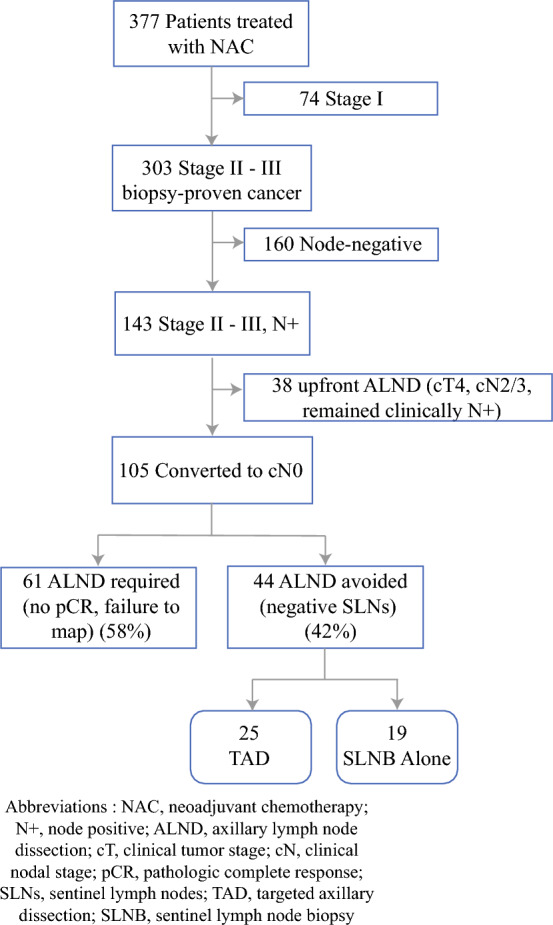
Study flowchart

## Methods

After institutional review board approval, consecutive patients with stage II or III, biopsy-proven node-positive breast cancer treated with NAC from August 2018 to June 2022 were identified from a prospective institutional database and electronic medical records. The study evaluated patients who became clinically node-negative after NAC (ycN0), defined as a negative physical exam of the axilla, or had tumor-free SLNB and avoided axillary lymph node dissection (ALND). The patients were divided into two groups: those treated with TAD and those who underwent SLNB alone.

At this institution, TAD was first used in August 2018. Use of TAD versus SLNB alone was surgeon-specific, and both methods were used during the entirety of the study period. Selection of tracer for SLNB was also surgeon-specific. Both isosulfan and methylene blue were used for blue dye, and technetium Tc-99M sulfur colloid was used as a nuclear tracer.

For the patients treated with TAD, a non-radioactive SAVI scout device (Cianna Medical, Inc, Aliso Viejo, CA, USA) or wire was placed to locate the clipped node. Axillary recurrence was monitored with review of patient symptoms, annual physical exam of the axilla, annual mammogram, and additional directed imaging as needed. Axillary ultrasound was not routinely used unless the patient had a specific complaint or a physical exam finding. Clinicopathologic data were collected from medical records.

The primary outcome variable included axillary nodal recurrence rates of TAD versus SLNB alone. Additional patient variables collected were age, menopausal status, self-reported ethnicity and race, tumor size, histologic findings, tumor differentiation, receptor status, type of operation performed, pCR, use of dual tracer, number of sentinel lymph nodes retrieved, neoadjuvant chemotherapy regimen, adjuvant chemotherapy, receipt of adjuvant radiotherapy, receipt of nodal radiotherapy, endocrine therapy, local recurrence, axillary recurrence, and distant recurrence.

Data are presented as frequency (%) for categorical variables and as median (interquartile range) for nonparametric continuous variables. Univariate associations between variables were examined with the Kruskal-Wallis test, chi-square test, or Fisher’s exact test where appropriate. All statistical analyses were performed using IBM SPSS, version 25.0 (SPSS, Inc, Armonk, NY) with two-sided tests and a significance level of 0.05.

## Results

Of the 377 patients treated with NAC, 143 (38%) had stage II or III node-positive breast cancer, and 105 (73%) were converted to ycN0. Of these 105 patients, 61 (58%) had an ALND due to tumor-involved nodes or failure to map, and 44 (42%) had tumor-free SLNB and avoided an ALND. Of the 44 patients, 25 (57%) had TAD, and 19 (43%) had SLNB alone.

The TAD and SLNB-alone groups were clinically similar. The median age was 49 years (interquartile range [IQR], 39–54 years). The majority of the patients were premenopausal (*n* = 25, 56.8%) and Caucasian (*n* = 26, 59.1%). The median tumor size at presentation was 2.7 cm (IQR, 1.9–3.4 cm). Most of the tumors were invasive ductal carcinoma (*n* = 43, 97.7%) and poorly differentiated (*n* = 33, 75.0%). As diagnosed, 25 (56.8%) of the tumors were human epidermal growth factor receptor 2 (HER2)-positive, 8 (18.2%) were hormone receptor-positive (HR+)/HER2–, and 11 (25%) were triple-negative. Breast-conserving surgery was performed for 18 (40.9%) patients, whereas mastectomy was administered to 26 (59.1%) patients, and 25 (56.8%) patients had breast pCR (TAD 60%, SLNB alone 52.6%; *p* = 0.633). The remaining patients had residual disease (Table 1).

**Table 1:** Clinicopathologic characteristics of the study cohort^a^

	All patients	TAD	SLNB alone	*p* Value
	(*n* = 44) *n* (%)	(*n* = 25) *n* (%)	(*n* = 19) *n* (%)	
Median age: years (IQR)	49 (39–54)	49 (41–55)	48 (38–53)	1.00
Menopausal status				0.90
Premenopausal	25 (56.8)	14 (56.0)	11 (57.9)	
Postmenopausal	19 (43.2)	11 (44.0)	8 (42.1)	
Race and ethnicity				0.91
Asian	6 (13.6)	3 (12.0)	3 (15.8)	
Black	3 (6.8)	2 (8.0)	1 (5.3)	
Hispanic	7 (15.9)	5 (20.0)	2 (10.5)	
Caucasian	26 (59.1)	14 (56.0)	12 (63.2)	
Other/unknown	2 (4.5)	1 (4.0)	1 (5.3)	
Median tumor size at presentation: cm (IQR)	2.7 (1.9–3.4)	2.7 (1.9–3.2)	2.9 (1.9–3.7)	0.792
Histologic findings				0.25
Ductal	43 (97.7)	25 (100)	18 (94.7)	
Lobular and mixed	0 (0)	0 (0)	0 (0)	
Occult	1 (2.3)	0 (0)	1 (5.3)	
Differentiation				0.19
Well	1 (2.3)	1 (4.0)	0 (0)	
Moderate	9 (20.5)	3 (12.0)	6 (31.6)	
Poor	33 (75.0)	21 (84.0)	12 (63.2)	
Unknown	1 (2.3)	0 (0)	1 (5.3)	
Receptor status				0.25
HR+/HER2–	8 (18.2)	4 (16.0)	4 (21.1)	
HR+/HER2+	14 (31.8)	6 (24.0)	8 (42.1)	
HR–/HER2+	11 (25)	6 (24.0)	5 (26.3)	
HR–/HER2–	11 (25)	9 (36.0)	2 (10.5)	
Breast surgery				0.63
Breast-conserving surgery	18 (40.9)	11 (44.0)	7 (36.8)	
Mastectomy	26 (59.1)	14 (56.0)	12 (63.2)	
Breast pCR (ypT0/Tis)	25 (56.8)	15 (60.0)	19 (52.6)	0.63
Dual tracer for SLNB				0.077
Yes	39 (88.6)	24 (96.0)	15 (78.9)	
No	5 (11.4)	1 (4.0)	4 (21.1)	
Median SLNs retrieved: *n* (IQR)	4 (3–8)	4 (3–9)	4 (2–8)	0.55
Biopsy-proven lymph node with clip excised				**0.03**
Yes	32 (72.7)	22 (88.0)	10 (52.6)	
No (clipped node retained)	5 (11.4)	1 (4.0)	4 (21.1)	
Unknown: biopsied node without clip (FNA) or no x-ray done	7 (15.9)	2 (8.0)	5 (26.3)	
Clipped node an SLN (blue or radioactive)				0.49
Yes	31 (96.9)	21 (95.5)	10 (100)	
No	1 (3.1)	1 (4.5)	0 (0)	

TAD, targeted axillary dissection; SLNB, sentinel lymph node biopsy; IQR, interquartile range; HR, hormone receptor; HER2, human epidermal growth factor receptor 2; pCR, pathologic complete response; SLN, sentinel lymph node

^a^Frequency (*n* [%]) reported for categorical variables and median (IQR) reported for continuous variables

The majority of the patients in each group had use of a dual tracer for SLNB (overall, 88.5% [39/44]; *p* = 0.077). Three patients had TAD using wire localization, and the remaining patients had SAVI scout localization. The median number of SLNs retrieved was 4 (IQR 3-8) (overall 4, TAD 4, SLNB alone 4; *p* = 0.55). The SLNB-alone approach was less likely to retrieve the biopsy-proven clipped node (clipped node retained: overall [*n* = 5/37], TAD [*n* = 1], SLNB alone [*n* = 4]; *p* = 0.03). The clipped node was a blue or radioactive sentinel lymph node for 21 (95.5%) patients in the TAD group and all the patients in the SLNB-alone group (*p* = 0.49; Table 1).

Doxorubicin-based NAC was administered to 21 (48%) patients. All the patients with HER2+ tumors received anti-HER2 neoadjuvant therapy and adjuvant therapy. For the adjuvant therapy, 19 (76.0%) of the patients received adjuvant trastuzumab and pertuzumab, and the remaining 6 (24.0%) patients received trastuzumab emtansine. The majority of the patients with triple-negative tumors (*n* = 7, 63.6%) received neoadjuvant pembrolizumab, and those who did not achieve a pCR received adjuvant capecitabine. Of the patients with HR+ tumors, 21 (95.5%) received adjuvant endocrine therapy with either an aromatase inhibitor or tamoxifen, 40 (91%) received adjuvant radiotherapy (RT), and 32 (73%) received regional nodal RT (Table 2).

**Table 2:** Treatment characteristics^a^

	No.	All patients *n* (%)	TAD *n* (%)	SLNB alone *n* (%)	*p* Value
NAC regimen	44				0.310
AC-T		20 (45.5)	13 (52.0)	7 (36.8)	
AC-T + carboplatin		1 (2.3)	1 (4.0)	0 (0)	
TC		16 (36.4)	9 (36.0)	7 (36.8)	
Other		7 (15.9)	2 (8.0)	5 (26.3)	
Neoadjuvant anti-HER2 therapy	25				n/a
HP		25 (100)	12 (100)	13 (100)	
Pembrolizumab given if TNBC	11	7 (63.6)	6 (66.7)	1 (50.0)	0.145
Adjuvant RT	44				0.441
Yes		40 (90.9)	22 (88.0)	18 (94.7)	
No		4 (9.1)	3 (12.0)	1 (5.3)	
Nodal RT	44				0.466
Yes		32 (72.7)	18 (72.0)	14 (73.7)	
No		11 (25.0)	7 (28.0)	4 (21.1)	
Unknown		1 (2.3)	0 (0)	1 (5.3)	
Adjuvant endocrine therapy	22				0.166
Yes		21(95.5)	9 (90.0)	12 (100)	
No		1 (4.5)	1 (10.0)	0 (0)	
Endocrine therapy regimen	22				0.310
Tamoxifen		7 (31.8)	3 (30.0)	4 (33.3)	
Aromatase inhibitor		14 (63.6)	6 (60.0)	8 (66.6)	
Unknown		1 (4.5)	1 (10.0)	0 (0)	
Adjuvant anti-HER2 therapy	25				0.283
Yes: HP		19 (76.0)	10 (83.3)	9 (69.2)	
Yes: TDM1		6 (24.0)	2 (16.6)	4 (30.8)	
No		0 (0)	0 (0)	0 (0)	
Adjuvant capecitabine if TNBC and no pCR	5				n/a
Yes		5 (100)	4 (100)	1 (100)	
No		0 (0)	0 (0)	0 (0)	

TAD, targeted axillary dissection; SLNB, sentinel lymph node biopsy; NAC, neoadjuvant chemotherapy; AC-T, adriamycin and cyclophosphamide followed by taxol; TC, Taxotere and cyclophosphamide; HER2, human epidermal growth factor receptor 2; HP, trastuzumab (H) and pertuzumab (P); RT, radiotherapy; TNBC, triple-negative breast cancer; RT, radiotherapy; TDM1, trastuzumab emtansine; pCR, pathologic complete response; IQR, interquartile range

^a^Frequency (*n* [%)] reported for categorical variables and median (IQR) reported for continuous variables

During a median follow-up period of 28 months (IQR, 21–40 months), there were no axillary nodal recurrences in either group. The majority of the patients (*n* = 41, 93.2 %) did not have a local recurrence. One patient in the TAD group and one patient in the SLNB-alone had a local skin recurrence. One patient in the TAD group had a local breast recurrence. The median time to local recurrence was 30 months. No patients had a chest wall or distant recurrence (Table 3**)**.

**Table 3:** Outcomes^a^

	All patients	TAD	SLNB alone	*p* Value
	(*n* = 44) *n* (%)	(*n* = 25) *n* (%)	(*n* = 19) *n* (%)	
Median follow-up: months (IQR)	28 (21–40)	21 (28–42)	28 (21–36)	1
Local recurrence				0.668
No	41 (93.2)	23 (92.0)	18 (94.7)	
Local (skin)	2 (4.5)	1 (4.0)	1 (5.3)	
Local (breast)	1 (2.3)	1 (4.0)	0 (0)	
Local (chest wall)	0 (0)	0 (0)	0 (0)	
Axillary recurrence				n/a
No	44 (100)	25 (100)	19 (100)	
Yes	0 (0)	0 (0)	0 (0)	
Distant recurrence	0 (0)	0 (0)	0 (0)	n/a
Median time to recurrence: months (IQR)	30 (6–40)	35 (30–40)	6 (6–6)	0.934

TAD, targeted axillary dissection; SLNB, sentinel lymph node biopsy; IQR, interquartile range; n/a

^a^Frequency (*n* [%]) reported for categorical variables and median (IQR) reported for continuous variables

## Discussion

The ACOSOG Z1071 study examined the false-negative rate (FNR) of SLNB for patients with node-positive breast cancer whose axilla was clinically downstaged after NAC.^1^ The study defined FNR as the frequency with which patients who had negative sentinel nodes were found to have metastatic disease in non-sentinel axillary nodes with subsequent ALND. The prespecified threshold for FNR was 10%, and the study showed a FNR of 12.6%. Use of dual-mapping with both a radioisotope/blue dye and retrieval of two or more sentinel lymph nodes lowered the FNR to 10.8% and 12.8%, respectively. The authors concluded that SLNB should not be used as an alternative to ALND at that time. Although it still did not meet the prespecified threshold, subsequent prospective trials SENTINA,^2^ SN FNAC,^3^ and GANEA 2^4^ demonstrated an acceptable FNR lower than 10 % with dual-mapping and three or more sentinel lymph nodes examined.

A meta-analysis of SLNB after NAC for patients with initial biopsy-proven node-positive breast cancer comprising 13 studies of 1921 patients demonstrated a pooled estimated FNR of 14%.^9^ In a subgroup analysis, the FNR with use of dual-agent mapping was 11% versus 19% with single-agent mapping. Furthermore, the FNR was 20% with removal of one node, 12% with removal of two nodes, and 4% with removal of three or more nodes. Similarly, use of dual-agent mapping and retrieval of three or more sentinel lymph nodes was predictive of the axilla for these patients. Although our institution used dual-mapping in the majority of both the TAD and SLNB-alone cases, increased use of dual-mapping in the SLNB-alone group would have helped the surgeons who did not perform TAD successfully to resect the clipped node as a sentinel node.

Another proposed method to decrease the FNR is clip placement in the node showing metastasis at the initial diagnosis, with confirmation of clipped-node resection at surgery. In an unplanned retrospective analysis from the ACOSOG Z1071 trial, in which the clipped node was within the sentinel lymph node specimen and two or more lymph nodes were retrieved, the FNR was 6.8%.^10^ Although this was a promising way to reduce FNR, the clip was present in an SLN in only 55 % of the patients, and thus would benefit only approximately half of patients.

Because of this limitation that the biopsy-proven clipped node may not be a sentinel lymph node and may be potentially retained, the technique of a targeted axillary dissection (TAD), including SLNB and selective localization with removal of the node with previously biopsy-proven metastasis, was developed.^6^ A prospective study from MD Anderson Cancer Center that investigated 208 patients with clinically node-positive breast cancer who had NAC and SLNB with selective removal of the clipped node using iodine-25 seed localization demonstrated an FNR of 4.2%.^6^ The limitations of this study were the single institution, the small sample size, and the possible dependency on the technique skill of the radiologist. Moreover, it is unclear whether the study is reproducible in a community setting. Notably, the clipped node was not retrieved as an SLN in 23% of the patients.

Since the Caudle et al. description of TAD, many other institutions have incorporated TAD into their practice In Germany, the SenTa trial investigated the feasibility and accuracy of wire-localized TAD across 50 centers with 548 patients and demonstrated a TAD success rate of 86.9% and an FNR rate of 7.2%.^11^ Worldwide, numerous institutions have implemented TAD^12^ with variations in technique.^13^ Siso et al.^14^ used intraoperative ultrasound to identify the ultrasound-visible marker in the clipped node after neoadjuvant chemotherapy for TAD. In Denmark, Munck et al. described placement of an iodine-25 seed before NAC, with a successful TAD for 91.5% of the patients (*n* = 142). Although axillary pCR rates of 40.8% were reported, axillary recurrence was not evaluated. Baker et al.^15^ described a pilot study of 23 patients from February 2019 to January 2020 in which a non-radioactive SAVI scout was used to locate the biopsy-proven metastasis clipped node before NAC in patients who had axillary downstaging. They were able to retrieve the SAVI scout identified in a lymph node from all the patients, but clip migration was observed in two patients. Their study demonstrated an axillary pCR rate of 44% and did not report axillary recurrence rates.

Our institution similarly began to use this technique the year before August 2018 with TAD using an SAVI scout or wire localization of the clipped node. Although the localization device was initially placed after chemotherapy, the localizations were technically difficult in cases in which the nodes clinically responded to NAC. Therefore, the protocol was changed to place them before neoadjuvant chemotherapy to ensure more accurate localization of the biopsy-proven node. Our cohort of patients included only those who had an axillary pCR because the patients who had a positive SLNB or TAD proceeded to ALND. Furthermore, our study not only described the TAD method, but also had a comparison group during the same study period for the patients who had SLNB alone to compare clinical outcomes and recurrence.

Although prior research shows the feasibility of the TAD technique, few studies have reported on recurrence outcomes. Barrio et al.^5^ demonstrated low axillary recurrence in patients with cN1 disease who converted to cN0 after NAC and had three or more negative SLNs with SLNB alone without routine nodal clipping. Among 234 patients, the authors reported one axillary recurrence in a patient who refused radiation during a median follow-up period of 40 months. They concluded that these findings potentially support omitting ALND for such patients. Notably, because they do not use TAD at this institution and did not always retrieve the clipped node, they still showed axillary recurrence to be low. Although they demonstrated that leaving biopsy-proven clipped nodes did not increase the risk of axillary recurrence, they did not have a comparison group for patients who had TAD.

The strength of our study was the comparison of patients who had SLNB alone and those who underwent TAD. In our study, both groups similarly showed no axillary recurrences at a median of 28 months despite a statistically significant higher clipped node retention in the SLNB-alone group.

Another benefit of retrieving the clipped node is the potential sparing of nodal radiation for those who achieve an axillary pCR. Three of the five patients who did not have the clipped node retrieved received nodal irradiation. It may be important to excise this clipped node because results of the NRG Oncology/NSABP B-51/RTOG 1304 trial evaluating invasive breast cancer recurrence in this population of patients with positive axillary nodes who are ypN0 after NAC become applicable.^16^

In the Netherlands, Nijveldt et al.^17^ implemented TAD procedures using radioactive I-125 seeds in 309 patients and showed during a median follow-up period of 2.8 years that 14% of the patients experienced recurrence, and only 3.5% had an axillary ecurrence. Although they similarly demonstrated that axillary recurrences were low in this group, they did not report a comparison group to evaluate outcomes for patients who did not have the clipped node retrieved.

Although data have emerged demonstrating low axillary recurrence with either TAD or SLNB alone, data on institutions comparing methods of TAD and SLNB alone are limited. Our study demonstrated low axillary recurrences for both the patients who had SLNB alone and those who had TAD despite a higher non-retrieval of the clipped node in the SLNB-alone group. This allowed for a more balanced comparison of patients during the same period, when medical treatments with chemotherapy and immunotherapy were similar.

Some limitations in applying TAD to the community setting must be considered. Availability of radiologists and surgeons who can perform axillary localizations before or after chemotherapy can be challenging because of difficulty identifying the clipped node. Also, the surgical technique has some limitations. Clips can migrate and be lost or suctioned out, and clips may not be in the actual lymph node. Localizations can lead to larger resections of axillary tissue, and the clipped node may not be retrieved despite the targeted effort.

Although patients may feel uneasy knowing that the clipped node with potentially unresected cancer is retained due to the limitations of the TAD identification and clip migration, the findings of this study show that axillary recurrence rates are low regardless of the technique. The American College of Surgeons (ACS) Commission on Cancer (CoC) operative standard 5.3 explains that although they do not require the clipped node be removed, whether it is removed or not is dictated in the operative synoptic report.^18^

The strengths of our study were the concurrent comparison of the techniques during the same period with modern systemic therapy. The limitation of this research was its small sample because we did not want to report patients treated with surgery before August 2018, prior to the time TAD was used at our institution, who had SLNB alone with different systemic management of their breast cancer, which could affect recurrence outcomes. The follow-up period was short, but most axillary recurrences are an early event. Although node-positive SLNB patients were not the focus of this study, the feasibility of TAD versus SLNB alone in this cohort should be further explored.

## Conclusions

For the patients with stage II or III node-positive breast cancer who became cN0 after NAC with tumor-free sentinel nodes, the axillary nodal recurrence rates were low after both TAD and SLNB alone despite higher non-retrieval of the clipped node in the SLNB-alone group. These findings suggest that either method affords excellent staging and regional control.
